# RNA-Mediated Regulation of HMGA1 Function

**DOI:** 10.3390/biom5020943

**Published:** 2015-05-14

**Authors:** Arndt G. Benecke, Sebastian Eilebrecht

**Affiliations:** 1 Université Pierre et Marie Curie, CNRS UMR 7224, 7 quai Saint Bernard, Paris 75005, France; 2 Deutsches Krebsforschungszentrum, Im Neuenheimer Feld 242, Heidelberg 69120, Germany

**Keywords:** HMGA1, non-coding RNA, 7SK RNA, HIV-1 TAR, transcription, chromatin

## Abstract

The high mobility group protein A1 (HMGA1) is a master regulator of chromatin structure mediating its major gene regulatory activity by direct interactions with A/T-rich DNA sequences located in the promoter and enhancer regions of a large variety of genes. HMGA1 DNA-binding through three AT-hook motifs results in an open chromatin structure and subsequently leads to changes in gene expression. Apart from its significant expression during development, HMGA1 is over-expressed in virtually every cancer, where HMGA1 expression levels correlate with tumor malignancy. The exogenous overexpression of HMGA1 can lead to malignant cell transformation, assigning the protein a key role during cancerogenesis. Recent studies have unveiled highly specific competitive interactions of HMGA1 with cellular and viral RNAs also through an AT-hook domain of the protein, significantly impacting the HMGA1-dependent gene expression. In this review, we discuss the structure and function of HMGA1-RNA complexes during transcription and epigenomic regulation and their implications in HMGA1-related diseases.

## 1. Introduction

**Figure 1:**
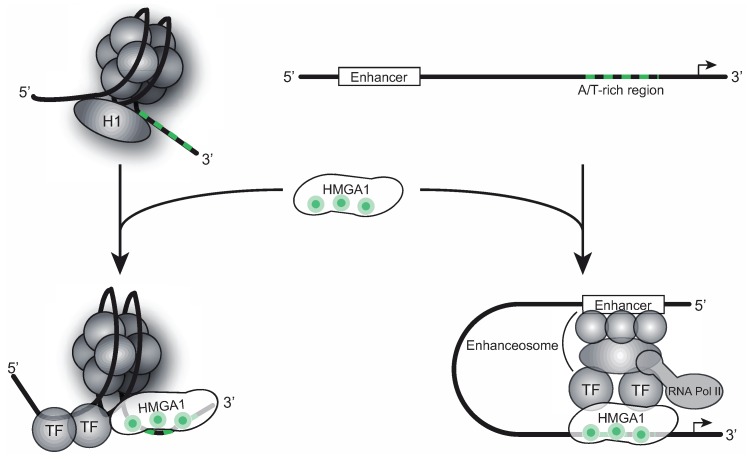
Chromatin- and gene expression regulation by HMGA1. HMGA1 binds to A/T-rich DNA sequences in gene promoter and enhancer regions. It acts as an antagonist of the linker histone H1, resulting in an open chromatin structure, permissive for gene transcription. Interacting with different transcription factors, HMGA1 is involved in enhanceosome formation, that way regulating gene-specific transcription.

**Figure 2:**
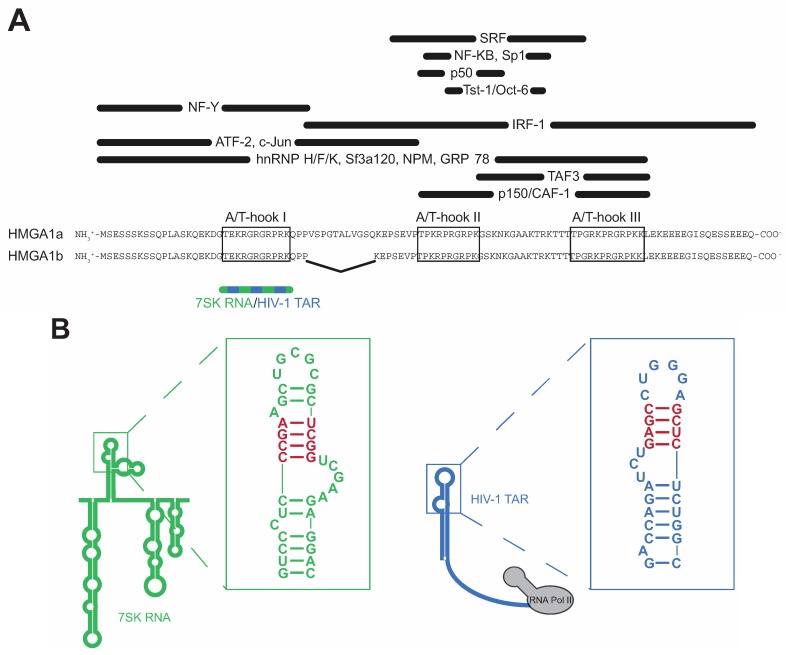
Schematic view of HMGA1 functional domains and RNA interfaces. (**A**) Schematic view of the HMGA1a/b functional domains. Interaction sites with transcription factors are labeled in black, the interface with 7SK and TAR RNA is depicted in blue/green; (**B**) Secondary structures of 7SK Loop2 (green) and HIV-1 TAR RNA (blue). The specific HMGA1-binding structures are highlighted in red.

HMGA1 belongs to the high mobility group (HMG) protein family, comprising a variety of non-histone proteins involved in global chromatin remodeling [1]. Within this family, the HMGA proteins are characterized by the presence of three AT-hook DNA binding motifs containing the core peptide Pro-Arg-Gly-Arg-Pro (P-R-G-R-P), allowing them to preferentially bind to the minor groove of A/T-rich B-form DNA sequences [2]. Though all three motifs synergize during target recognition, the first two AT-hooks contribute the majority of HMGA1’s DNA affinity [3]. HMGA1 proteins act as antagonists of the linker histone H1, which binds to the same DNA sequences and maintains chromatin in a tightly packed, transcription-inactive state [4]. Thus, HMGA1 proteins introduce major changes in DNA structure, resulting in a more open chromatin state, which facilitates gene transcription (Figure 1). Apart from this global role as master regulators of chromatin structure, HMGA1 proteins physically interact with a large variety of different transcription factors, such as Sp1, NF-κB, NF-Y, ATF-2, c-Jun, TAF3, p150 and others [5,6,7,8], orchestrating their assembly at gene promoter and enhancer regions, also assigning them important functions during gene-specific transcription regulation (Figure 1). The HMGA1 gene encodes for two alternatively spliced isoforms HMGA1a and HMGA1b, the latter one lacking 11 amino acids between the first and the second AT-hook motif [9,10] (Figure 2A).

HMGA1 proteins are typically highly expressed during development where several studies assign them important roles in regulating normal cell proliferation, embryonic cell growth and cell differentiation [11,12,13,14]. However, after early embryonic development, HMGA1 expression drops to low or undetectable levels in differentiated adult tissues or non-proliferating cells [12,15]. Remarkably, HMGA1 proteins are over-expressed in virtually every type of cancer, where their expression levels correlate with tumor malignancy and a poor outcome for patients suffering from that particular type of tumor (reviewed in [16]). Moreover, the induced overexpression of HMGA1 in immune-inactivated nude mice leads to malignant tumor formation and HMGA1 expression also correlates with the metastatic potential of the tumor [17,18], making HMGA1 a key player during cancerogenesis. Due to its reliably high expression in almost every type of malignant tumor, HMGA1 is increasingly put forward as a novel marker for medical prognosis. Apart from its roles during tumorigenesis, HMGA1 has been shown to be involved in gene expression regulation of several types of viruses, including human papovavirus JC [8], Epstein-Barr Virus (EBV) [19], Herpes Simplex Virus (HSV-1) [20,21] and Human Immunodeficiency Virus (HIV-1) [22].

In recent studies, we have identified highly specific interactions of HMGA1a protein with the nuclear non-coding 7SK RNA and the transactivating response element (TAR) located in the nascent transcript of HIV-1 [23,24,25]. 7SK RNA is a highly abundant RNA Polymerase III transcript in eukaryotic cells, which is a negative regulator of RNA Polymerase II transcription elongation by inactivating the Positive Transcription Elongation Factor b (P-TEFb) [26,27,28,29]. Thereby, 7SK RNA acts as a scaffold, which mediates the interaction of P-TEFb with its inhibitors HEXIM1 and CTIP2, resulting in P-TEFb inactivation [30,31]. HIV-1 TAR is a RNA secondary structure formed by the nascent viral transcript, which is involved in viral transcription activation by recruiting HIV-1 Tat-bound P-TEFb to the promoter proximal paused host cellular RNA Polymerase II. This review focusses on the structure and function of these HMGA1-RNA complexes as well as their implications in HMGA1-related diseases.

## 2. HMGA1-RNA Interactions from the Structural Point of View

While the interaction of HMGA1 with DNA via its three AT-hook motifs has been extensively studied during the last decades [32,33], RNA-HMGA1 interactions have been identified only very recently and to date, detailed structural studies are still lacking. However, a reasonable number of analyses aimed at deciphering the RNA-HMGA1 interface, gaining insights into the structural basis of these interactions.

In 2007, a study by Manabe and colleagues pointed at a role of HMGA1 during exon-skipping of presenilin-2 pre-mRNA, which results in the production of a deleterious protein isoform found in brains of patients suffering from Alzheimer’s disease, and thus provided first evidence for a specific RNA affinity of HMGA1 [34]. In more recent studies, we identified the RNA Polymerase III-transcribed non-coding 7SK RNA as a novel HMGA1 binding partner [23,24]. 7SK RNA is a highly abundant housekeeping RNA located in the nucleus of eukaryotic cells [35]. It has been identified as a key negative regulator of global RNA Polymerase II transcription elongation reaction by inactivating the positive transcription elongation factor b (P-TEFb), whose active form phosphorylates the carboxy-terminal domain (CTD) of RNA Polymerase II in order to start the efficient transcription elongation reaction [26,27,28,29]. Thereby, 7SK RNA acts as a scaffold, which mediates close spatial proximity of the negative P-TEFb regulators HEXIM1 and CTIP2 and P-TEFb itself, resulting in P-TEFb inactivation [30,31]. On demand (e.g., upon stress signaling), P-TEFb is released from this complex, becomes activated and thus is able to catalyze the transcription elongation reaction. The interaction of HMGA1 with 7SK RNA is highly specific for both sites: HMGA1 specifically recognizes the second major hairpin (loop 2) of 7SK RNA by its first, N-terminally located AT-hook motif [23] (Figure 2). Thereby, a stem structure of 7SK RNA consisting of three G-C base pairs and one A-U base pair following a bulge constitutes the core motif for HMGA1 binding (Figure 2B, left, red). Mutation studies point at the tertiary structure of this region to play a major role for HMGA1 recognition rather than the secondary structure or RNA sequence itself [23].

Aside from the interaction with 7SK RNA, we have recently shown that HMGA1 also specifically binds a RNA structure located in the nascent transcript of the HIV-1 genome, known as the transactivating response element (HIV-1 TAR) [25]. HIV-1 transcription is regulated by promoter-proximal pausing of RNA Polymerase II and thus strongly depends on active P-TEFb (reviewed in [36]). HIV-1 TAR thereby is essential by mediating the recruitment of HIV-1 Tat-bound, active P-TEFb to the promoter proximal paused RNA Polymerase II. During this process, the secondary structure of HIV-1 TAR is recognized by the viral transactivator of transcription (Tat), which is able to eject P-TEFb from the inactive 7SK/HEXIM1 complex, resulting in P-TEFb activation [37]. Also, in the case of HIV-1 TAR, HMGA1 recognizes a hairpin structure containing a stem of three G-C base pairs and one A-U base pair following a bulge structure (Figure 2B, right, red). Remarkably, this region overlaps with the binding interface for HIV-1 Tat [38] and competitive binding assays have proven incompatibility between HMGA1 and HIV-1 Tat for concomitant TAR binding [25]. Also, in the case of HIV-1 TAR recognition, the first, N-terminally located AT-hook motif of HMGA1 is the site of interaction.

## 3. HMGA1-Regulation by 7SK RNA

The fact that one of the high-affinity DNA-binding domains of HMGA1 (the first AT-hook motif) is the binding site for 7SK RNA suggests a role of 7SK RNA as a competitive regulator of those HMGA1 functions necessitating a direct interaction of HMGA1 with DNA, such as chromatin regulation and enhanceosome formation (see Figure 1).

**Figure 3:**
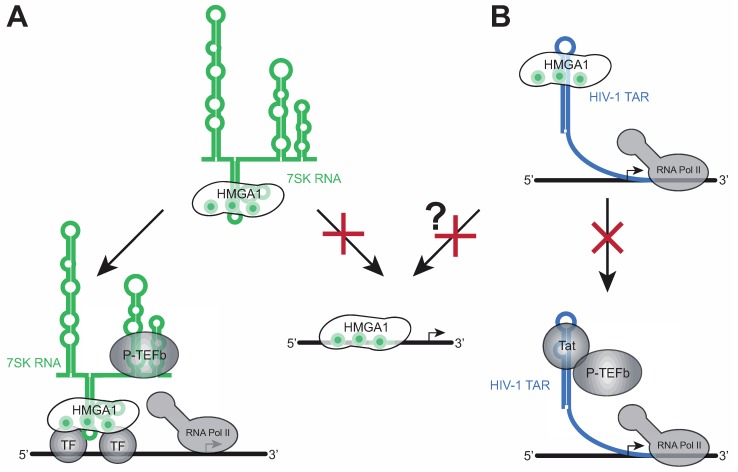
Cellular functions of HMGA1-RNA complexes. (**A**) HMGA1 is involved in the recruitment of 7SK-inactivated P-TEFb to the HIV-1 and cellular core promoters. 7SK RNA acts as a negative regulator of HMGA1 DNA-binding, subsequently regulating HMGA1 target gene expression; (**B**) The HMGA1/TAR interaction prevents Tat/TAR binding. HIV-1 TAR might also negatively regulate HMGA1-dependent gene expression.

Indeed, the overexpression of the 7SK loop 2 substructure as a chimera with the EBER2 RNA of Epstein-Barr Virus in a cell culture model, taking advantage of the strong EBER2 promoter [39,40], leads to changes in gene expression, which are almost identical to those upon a knockdown of HMGA1, supporting a negative regulatory effect of 7SK RNA on HMGA1 function [23]. Furthermore, 7SK RNA was proven to compete with DNA (e.g., the promoter sequence of the direct HMGA1 target IL-2Rα [41,42]) for HMGA1 binding [23], indicating 7SK RNA to primarily affect HMGA1 functions involving direct HMGA1/DNA interactions (Figure 3A, right arrow). The overexpression approach of the HMGA1-binding 7SK loop 2 substructure has proven to efficiently target HMGA1-responsive genes, such as MAP2K2, IGFBP2, SOX4, GNAZ, STK6, CCND3, LAMA1, ACSL3, ARL3, COL6A1, COX2, IFNAR and HLA-A, making the EBER2-7SK L2 fusion construct a useful tool to regulate HMGA1 activity, when using the EBER2 backbone as a control [23]. Given the strong overexpression of HMGA1 in the vast majority of cancers and its causal effects during malignant transformation, the negative regulation of HMGA1 function by overexpressing 7SK Loop2 RNA or full length 7SK RNA might serve as a reasonable alternative or complement for HMGA1-targeting anticancer therapeutic approaches such as small interfering (si)RNA-mediated HMGA1 knockdown [16].

## 4. HMGA1 and 7SK-Dependent Transcription Elongation

The first AT-hook motif of HMGA1 contributes the majority of HMGA1’s DNA binding capacity [3]. Binding of the 7SK Loop2 RNA to exactly this site competes with HMGA1 at its DNA target sites, resulting in strong changes in HMGA1-dependent gene expression [23,24]. However, to date, it remains elusive, whether the 7SK-HMGA1 interaction also impacts HMGA1’s binding to a large variety of transcription factors, but the interfaces for the majority of these factors do not overlap with the first AT-hook motif, suggesting that 7SK RNA does not directly influence their binding (Figure 2A). Even for those transcription factors whose HMGA1 interface includes the first AT-hook motif, it is not clear, whether 7SK RNA bound to HMGA1 enhances or decreases the binding, opening up the possibility that HMGA1 can recruit 7SK RNA and thereby the inactive P-TEFb complex to gene promoters via DNA-bound transcription factors (see Figure 3A, left).

In fact, we have shown a gene-specific cooperation of HMGA1 and P-TEFb during gene expression regulation [43]. Importantly, two preconditions for a HMGA1-mediated P-TEFb recruitment are fulfilled: The interaction of 7SK RNA with P-TEFb is mediated by the Loop1, 3 and 4 substructures of the RNA, while HMGA1 specifically binds the Loop2 substructure and co-immunoprecipitation studies prove that P-TEFb co-purifies with HMGA1 in a RNA-dependent manner [43].

In the case of HIV-1 transcription, the 7SK/P-TEFb complex has been shown previously to be located in close proximity to the HIV-1 core promoter, but a mechanism for its recruitment remained unexplored [44]. Subsequently the viral transactivator of transcription (Tat) replaces the inactivating compounds HEXIM1 and 7SK RNA and finally recruits the activated P-TEFb to the paused RNA Polymerase II by interacting with HIV-1 TAR (see Figure 3B, lower panel). In a recent study, we have uncovered a role of HMGA1 during the recruitment of the inactive P-TEFb complex to the HIV-1 core promoter [44]. We have reported on an interaction of HMGA1 with the P-TEFb inactivator CTIP2. ChIP analyses have revealed that both proteins are important during P-TEFb recruitment to the HIV-1 core promoter and several cellular promoters [44]. Given the large number of viruses, whose transcription is responsive to HMGA1, future studies will be required in order to investigate, whether a similar mechanism applies to other viruses as well.

The 7SK/HMGA1 interaction not only regulates HMGA1 function, but is also essential for the recruitment of 7SK-bound P-TEFb to cellular and viral promoters, establishing plasticity between HMGA1-dependent chromatin remodeling and P-TEFb-dependent transcription elongation regulation.

## 5. HMGA1-RNA Complexes during HIV-1 Transcription

HMGA1 has been previously implicated in HIV-1 infection as a host cellular co-factor recruiting the nucleosome remodeling complex SWI/SNF to the HIV-1 LTR, being involved in HIV-1 splice site regulation and contributing to the HIV-1 pre-integration complex [45,46,47,48,49,50]. Our findings of a specific interaction of HMGA1 with HIV-1 TAR, thereby competing with HIV-1 Tat for TAR-binding, add another facet to viral transcription regulation [25] (Figure 3B). While the Tat/TAR interaction is prerequisite for efficient viral transcription elongation, assigning HMGA1 a silencing role in HIV-1 gene expression, HIV-1 TAR might also function as a negative regulator of HMGA1 function, similar to 7SK Loop2 RNA, thereby regulating the expression of HMGA1 target genes.

Interestingly, our recent findings of HMGA1 being a recruiting factor for CTIP2-inactivated P-TEFb to the HIV-1 core promoter constitute another mechanism of HMGA1-mediated HIV-1 silencing [46,47]. Whether a similar pathway may also be involved in HMGA1-mediated gene expression regulation of the human papovavirus JC, EBV and HSV-1 will need to be addressed in future research. Note that apparent contradictions in the role of HMGA1 during HIV-1 transcription regulation such as the fact that HMGA1 complexes facilitate HIV-1 transcription through the recruitment of chromatin modifiers but also prevent HIV-1 reactivation through the inhibition of the elongation complex P-TEFb should be seen in light of the time, cell-specificity (e.g., co-factor expression), and multiple possibilities for post-translational modifications [47].

Taken together, HMGA1 depicts a host cellular co-factor involved in a large variety of aspects during HIV-1 infection, some of which may synergize, especially those involved in HIV-1 transcription regulation.

## 6. Conclusions

Abnormally high expression rates of HMGA1 constitute a hallmark for the vast majority of cancers (see Table 1) and generally correlate with a poor outcome [51]. Recent studies provide evidence for HMGA1 being a driving force during cancer growth and tumor progression [52,53]. By epigenetic reprogramming and regulation of gene networks—comprising OCT4 and cMYC—HMGA1 has been shown to maintain a pluripotent, undifferentiated state in embryonic stem cells, a mechanism by which the protein likely also contributes to cancerogenesis and tumor growth [53].

**Table 1:** HMGA1 association with human diseases.

Disease	HMGA1 Expression/Function	References
Bladder cancer	Overexpression	[54]
Breast cancer	Overexpression	[55,56,57]
Colorectal cancer	Overexpression; positively regulates Wnt/β-catenin signaling	[52,58]
Head and neck cancer	Overexpression	[59]
Leukemia	Overexpression; Cmyc target	[17,60,61]
Kidney cancer	Overexpression	[62]
Liver cancer	Overexpression	[63]
Lung cancer	Overexpression; promotes transformation	[64,65]
Glioblastoma/Neuroblastoma	Overexpression	[66,67,68,69]
Pancreatic cancer	Overexpression; promotes cellular invasiveness and metastatic potential	[70,71,72]
Prostate cancer	Overexpression; involved in chromosomal re-arrangements	[73,74]
Gastric cancer	Overexpression; let7-downregulation	[75,76]
Thyroid cancer	Overexpression; regulates expression of miR-603 and miR-10b	[77,78]
Cervix cancer	Overexpression	[79]
HIV infection	Co-factor for integration, transcription and spli-cing	[25,45,46,47,48,49,50]
Human papovavirus JC infection	Co-factor for transcription	[8]
Epstein Barr virus infection	Co-factor for transcription	[19]
Herpes Simplex virus 1 infection	Co-factor for transcription	[20,21]
Alzheimer’s disease	Involved in presenilin-2 pre-mRNA exon-skipping	[34]

Thus, targeting HMGA1 is increasingly gaining attention in the field of anti-cancer therapeutical approaches (reviewed in [16]). Recent studies aiming at silencing HMGA1 expression by delivery of short hairpin (sh)RNAs reported an attenuated growth and major changes in appearance of breast cancer cells upon HMGA1 knockdown [55]. In addition, tumorigenic properties such as cell mobility, invasion and anchorage-independent cell growth were diminished, making the HMGA1 knockdown a promising approach for fighting HMGA1-dependent cancer formation. A large variety of different compounds have been tested, which affect or inhibit HMGA1 binding to the minor groove of A/T-rich DNA regions. The crosslinking compounds FR900482 and FR66979 covalently link HMGA1 and its DNA target sequences, resulting in inhibited proliferation of T-cell acute lymphoblastic leukemia cells, but clinical trials had to be aborted due to unacceptable side effects [80,81,82]. The antibiotics distamycin and netropsin interfere with HMGA1-DNA binding by blocking the minor groove of A/T-rich DNA regions and are able to impact the expression of HMGA1 target genes [83,84]. However, minor groove DNA blocking agents do not specifically target HMGA1 function, as they also interfere with other minor groove binding factors. Another promising approach for targeting HMGA1 function is the use of A/T-rich oligonucleotides in order to sequester HMGA1 and prevent target binding, which resulted in tumor size reduction in xenograft tumors originating from cultured pancreatic adenocarcinoma cells [85,86].

Targeting HMGA1 function in gene expression regulation by 7SK Loop2 RNA, as demonstrated in our studies, may establish another promising approach for fighting HMGA1-dependent cancerogenesis [23,24]. The overexpression or delivery of a RNA structure originating from an endogenous housekeeping RNA thereby would likely minimize unwanted side effects. Though off-target effects induced by the Epstein-Barr viral EBER2 RNA backbone used here for 7SK Loop2 RNA overexpression will have to be considered carefully [87], recent RNA-based therapeutic approaches such as the targeted delivery of small RNA molecules or more stable locked nucleic acids (LNA) could allow the application of a backbone-free 7SK Loop2 RNA [88,89]. Targeting HMGA1 function would also be an interesting scenario in other diseases HMGA1 has directly been implicated in, such as cardiac hypertrophy [90], sepsis [91,92], type-2 diabetes [93], and the inflammatory response [92].

In the case of HIV-1 infection, HMGA1 affects viral gene expression by at least two different mechanisms: A direct binding to HIV-1 TAR, preventing Tat-mediated transcription activation and the recruitment of CTIP2 and CTIP2-inactivated P-TEFb complex [25,45]. Both pathways result in HIV-1 silencing and may contribute to viral latency, which is a major obstacle during highly active anti-retroviral therapy (HAART), which only targets cells actively transcribing HIV (reviewed in [94,95,96]). Also a potential regulation of HMGA1 target genes by HIV-1 TAR—as it is the case for 7SK Loop2—will have to be taken into account as an additional mechanism of HIV-mediated reprogramming of the host cell. 7SK Loop2 RNA has been shown to compete with HIV-1 TAR for HMGA1 binding [25]. Thus, also in the case of HIV-1 infection, the targeting of HMGA1 function by 7SK Loop2 could contribute to the reactivation of latent HIV-1 reservoirs, which could subsequently be eradicated by HAART.

Targeting HMGA1 function is also challenged by the fact that HMGA1 asides from its crucial role during development also is responsible for gene regulatory activity on cellular promoters during homeostasis. Indeed, HMGA1 is required for physiological expression of the insulin receptor [97] and glucose-induced insulin transcription [98]. Protecting these physiological functions while fighting disease needs major attention during the development of HMGA1 targeting strategies. Such strategies might therefore need to involve concomitant targeting of other protein components of the HMGA1 complexes such as P-TEFb or CTIP2 [99]. Yet, another complication to the targeting of HMGA1 function arises through the potential interference associated with the possible expression of HMGA1 pseudogenes [100].

Taken together, HMGA1 has been proven to be a key player during cancerogenesis and an important host cellular factor for the expression of a variety of viral genomes. Targeting HMGA1 function by an overexpression or delivery of the Loop2 region of its endogenous interaction partner 7SK RNA may thus add to a growing number of different approaches for fighting HMGA1-related diseases.

## References

[1] Reeves R., Beckerbauer L. (2001). HMGI/Y proteins: Flexible regulators of transcription and chromatin structure. Biochim. Biophys. Acta.

[2] Huth J.R., Bewley C.A., Nissen M.S., Evans J.N., Reeves R., Gronenborn A.M., Clore G.M. (1997). The solution structure of an HMG-I(Y)-DNA complex defines a new architectural minor groove binding motif. Nat. Struct. Biol..

[3] Harrer M., Lührs H., Bustin M., Scheer U., Hock R. (2004). Dynamic interaction of HMGA1a proteins with chromatin. J. Cell Sci..

[4] Krech A.B., Wulff D., Grasser K.D., Feix G. (1999). Plant chromosomal HMGI/Y proteins and histone H1 exhibit a protein domain of common origin. Gene.

[5] Yie J., Liang S., Merika M., Thanos D. (1997). Intra- and intermolecular cooperative binding of high-mobility-group protein I(Y) to the beta-interferon promoter. Mol. Cell. Biol..

[6] Currie R.A. (1997). Functional interaction between the DNA binding subunit trimerization domain of NF-Y and the high mobility group protein HMG-I(Y). J. Biol. Chem..

[7] Zhang X.M., Verdine G.L. (1999). A small region in HMG I(Y) is critical for cooperation with NF-κB on DNA. J. Biol. Chem..

[8] Leger H., Sock E., Renner K., Grummt F., Wegner M. (1995). Functional interaction between the POU domain protein Tst-1/Oct-6 and the high-mobility-group protein HMG-I/Y. Mol. Cell. Biol..

[9] Friedmann M., Holth L.T., Zoghbi H.Y., Reeves R. (1993). Organization, inducible-expression and chromosome localization of the human HMG-I(Y) nonhistone protein gene. Nucleic Acids Res..

[10] Nagpal S., Ghosn C., DiSepio D., Molina Y., Sutter M., Klein E.S., Chandraratna R.A. (1999). Retinoid-dependent recruitment of a histone H1 displacement activity by retinoic acid receptor. J. Biol. Chem..

[11] Chiappetta G., Avantaggiato V., Visconti R., Fedele M., Battista S., Trapasso F., Merciai B.M., Fidanza V., Giancotti V., Santoro M. (1996). High level expression of the HMGI (Y) gene during embryonic development. Oncogene.

[12] Bustin M., Reeves R. (1996). High-mobility-group chromosomal proteins: Architectural components that facilitate chromatin function. Prog. Nucleic Acids Res. Mol. Biol..

[13] Beaujean N., Bouniol-Baly C., Monod C., Kissa K., Jullien D., Aulner N., Amirand C., Debey P., Käs E. (2000). Induction of early transcription in one-cell mouse embryos by microinjection of the nonhistone chromosomal protein HMG-I. Dev. Biol..

[14] Melillo R.M., Pierantoni G.M., Scala S., Battista S., Fedele M., Stella A., de Biasio M.C., Chiappetta G., Fidanza V., Condorelli G. (2001). Critical role of the HMGI(Y) proteins in adipocytic cell growth and differentiation. Mol. Cell. Biol..

[15] Lundberg K., Karlson J.R., Ingebrigtsen K., Holtlund J., Lund T., Laland S.G. (1989). On the presence of the chromosomal proteins HMG I and HMG Y in rat organs. Biochim. Biophys. Acta.

[16] Huso T.H., Resar L. (2014). The high mobility group A1 molecular switch: Turning on cancer—Can we turn it off?. Expert Opin. Ther. Targets.

[17] Wood L.J., Mukherjee M., Dolde C.E., Xu Y., Maher J.F., Bunton T.E., Williams J.B., Resar L.M. (2000). HMG-I/Y, a new c-Myc target gene and potential oncogene. Mol. Cell. Biol..

[18] Reeves R., Edberg D.D., Li Y. (2001). Architectural transcription factor HMGI(Y) promotes tumor progression and mesenchymal transition of human epithelial cells. Mol. Cell. Biol..

[19] Schaefer B.C., Paulson E., Strominger J.L., Speck S.H. (1997). Constitutive activation of Epstein-Barr virus (EBV) nuclear antigen 1 gene transcription by IRF1 and IRF2 during restricted EBV latency. Mol. Cell. Biol..

[20] Panagiotidis C.A., Silverstein S.J. (1999). The host-cell architectural protein HMG I(Y) modulates binding of herpes simplex virus type 1 ICP4 to its cognate promoter. Virology.

[21] French S.W., Schmidt M.C., Glorioso J.C. (1996). Involvement of a high-mobility-group protein in the transcriptional activity of herpes simplex virus latency-active promoter 2. Mol. Cell. Biol..

[22] Henderson A., Bunce M., Siddon N., Reeves R., Tremethick D.J. (2000). High-mobility-group protein I can modulate binding of transcription factors to the U5 region of the human immunodeficiency virus type 1 proviral promoter. J. Virol..

[23] Eilebrecht S., Brysbaert G., Wegert T., Urlaub H., Benecke B.J., Benecke A. (2011). 7SK small nuclear RNA directly affects HMGA1 function in transcription regulation. Nucleic Acids Res..

[24] Eilebrecht S., Bécavin C., Léger H., Benecke B.J., Benecke A. (2011). HMGA1-dependent and independent 7SK RNA gene regulatory activity. RNA Biol..

[25] Eilebrecht S., Wilhelm E., Benecke B.J., Bell B., Benecke A.G. (2013). HMGA1 directly interacts with TAR to modulate basal and Tat-dependent HIV transcription. RNA Biol..

[26] Nguyen V.T., Kiss T., Michels A.A., Bensaude O. (2001). 7SK small nuclear RNA binds to and inhibits the activity of CDK9/cyclin T complexes. Nature.

[27] Yang Z., Zhu Q., Luo K., Zhou Q. (2001). The 7SK small nuclear RNA inhibits the CDK9/cyclin T1 kinase to control transcription. Nature.

[28] Zhou Q., Li T., Price D.H. (2012). RNA polymerase II elongation control. Annu. Rev. Biochem..

[29] Peterlin B.M., Brogie J.E., Price D.H. (2012). 7SK snRNA: A noncoding RNA that plays a major role in regulating eukaryotic transcription. Wiley Interdiscip. Rev. RNA.

[30] Yik J.H., Chen R., Nishimura R., Jennings J.L., Link A.J., Zhou Q. (2003). Inhibition of P-TEFb (CDK9/Cyclin T) kinase and RNA polymerase II transcription by the coordinated actions of HEXIM1 and 7SK snRNA. Mol. Cell.

[31] Cherrier T., le Douce V., Eilebrecht S., Riclet R., Marban C., Dequiedt F., Goumon Y., Paillart J.C., Mericskay M., Parlakian A. (2013). CTIP2 is a negative regulator of P-TEFb. Proc. Natl. Acad. Sci. USA.

[32] Watanabe M., Ni S., Lindenberger A.L., Cho J., Tinch S.L., Kennedy M.A. (2013). Characterization of the stoichiometry of HMGA1/DNA complexes. Open Biochem. J..

[33] Fonfría-Subirós E., Acosta-Reyes F., Saperas N., Pous J., Subirana J.A., Campos J.L. (2012). Crystal structure of a complex of DNA with one AT-hook of HMGA1. PLoS ONE.

[34] Manabe T., Ohe K., Katayama T., Matsuzaki S., Yanagita T., Okuda H., Bando Y., Imaizumi K., Reeves R., Tohyama M. (2007). HMGA1a: Sequence-specific RNA-binding factor causing sporadic Alzheimer’s disease-linked exon skipping of presenilin-2 pre-mRNA. Genes Cells.

[35] Gurney T., Eliceiri G.L. (1980). Intracellular distribution of low molecular weight RNA species in HeLa cells. J. Cell Biol..

[36] Ott M., Geyer M., Zhou Q. (2011). The control of HIV transcription: Keeping RNA polymerase II on track. Cell Host Microbe.

[37] Muniz L., Egloff S., Ughy B., Jády B.E., Kiss T. (2010). Controlling cellular P-TEFb activity by the HIV-1 transcriptional transactivator Tat. PLoS Pathog..

[38] Berkhout B., Jeang K.T. (1989). Trans activation of human immunodeficiency virus type 1 is sequence specific for both the single-stranded bulge and loop of the trans-acting-responsive hairpin: A quantitative analysis. J. Virol..

[39] Dumpelmann E., Mittendorf H., Benecke B.J. (2003). Efficient transcription of the EBER2 gene depends on the structural integrity of the RNA. RNA.

[40] Lerner M.R., Andrews N.C., Miller G., Steitz J.A. (1981). Two small RNAs encoded by Epstein-Barr virus and complexed with protein are precipitated by antibodies from patients with systemic lupus erythematosus. Proc. Natl. Acad. Sci. USA.

[41] Reeves R., Elton T.S., Nissen M.S., Lehn D., Johnson K.R. (1987). Posttranscriptional gene regulation and specific binding of the nonhistone protein HMG-I by the 3' untranslated region of bovine interleukin 2 cDNA. Proc. Natl. Acad. Sci. USA.

[42] Reeves R., Leonard W.J., Nissen M.S. (2000). Binding of HMG-I(Y) imparts architectural specificity to a positioned nucleosome on the promoter of the human interleukin-2 receptor alpha gene. Mol. Cell. Biol..

[43] Eilebrecht S., Benecke B.J., Benecke A. (2011). 7SK snRNA-mediated, gene-specific cooperativity of HMGA1 and P-TEFb. RNA Biol..

[44] D’Orso I., Frankel A.D. (2010). RNA-mediated displacement of an inhibitory snRNP complex activates transcription elongation. Nat. Struct. Mol. Biol..

[45] Henderson A., Holloway A., Reeves R., Tremethick D.J. (2004). Recruitment of SWI/SNF to the human immunodeficiency virus type 1 promoter. Mol. Cell. Biol..

[46] Eilebrecht S., le Douce V., Riclet R., Targat B., Hallay H., van Driessche B., Schwartz C., Robette G., van Lint C., Rohr O. (2014). HMGA1 recruits CTIP2-repressed P-TEFb to the HIV-1 and cellular target promoters. Nucleic Acids Res..

[47] Benecke A. (2003). Genomic plasticity and information processing by transcription coregulators. Complexus.

[48] Tsuruno C., Ohe K., Kuramitsu M., Kohma T., Takahama Y., Hamaguchi Y., Hamaguchi I., Okuma K. (2011). HMGA1a is involved in specific splice site regulation of human immunodeficiency virus type 1. Biochem. Biophys. Res. Commun..

[49] Gao K., Gorelick R.J., Johnson D.G., Bushman F. (2003). Cofactors for human immunodeficiency virus type 1 cDNA integration *in vitro*. J. Virol..

[50] Van Maele B., Busschots K., Vandekerckhove L., Christ F., Debyser Z. (2006). Cellular co-factors of HIV-1 integration. Trends Biochem. Sci..

[51] Fusco A., Fedele M. (2007). Roles of HMGA proteins in cancer. Nat. Rev. Cancer.

[52] Belton A., Gabrovsky A., Bae Y.K., Reeves R., Iacobuzio-Donahue C., Huso D.L., Resar L.M. (2012). HMGA1 induces intestinal polyposis in transgenic mice and drives tumor progression and stem cell properties in colon cancer cells. PloS ONE.

[53] Shah S.N., Resar L.M. (2012). High mobility group A1 and cancer: Potential biomarker and therapeutic target. Histol. Histopathol..

[54] Ben-Porath I., Thomson M.W., Carey V.J., Ge R., Bell G.W., Regev A., Weinberg R.A. (2008). An embryonic stem cell-like gene expression signature in poorly differentiated aggressive human tumors. Nat. Genet..

[55] Shah S.N., Cope L., Poh W., Belton A., Roy S., Talbot C.C., Sukumar S., Huso D.L., Resar L.M. (2013). HMGA1: A master regulator of tumor progression in triple-negative breast cancer cells. PloS ONE.

[56] Dolde C.E., Mukherjee M., Cho C., Resar L.M. (2002). HMG-I/Y in human breast cancer cell lines. Breast Cancer Res. Treat..

[57] Flohr A.M., Rogalla P., Bonk U., Puettmann B., Buerger H., Gohla G., Packeisen J., Wosniok W., Loeschke S., Bullerdiek J. (2003). High mobility group protein HMGA1 expression in breast cancer reveals a positive correlation with tumour grade. Histol. Histopathol..

[58] Xing J., Cao G., Fu C. (2014). HMGA1 interacts with β-catenin to positively regulate Wnt/β-catenin signaling in colorectal cancer cells. Pathol. Oncol. Res..

[59] Rho Y.S., Lim Y.C., Park I.S., Kim J.H., Ahn H.Y., Cho S.J., Shin H.S. (2007). High mobility group HMGI(Y) protein expression in head and neck squamous cell carcinoma. Acta Otolaryngol..

[60] Hillion J., Dhara S., Sumter T.F., Mukherjee M., di Cello F., Belton A., Turkson J., Jaganathan S., Cheng L., Ye Z. (2008). The High-mobility group A1a/signal transducer and activator of transcription-3 Axis: An achilles heel for hematopoietic malignancies?. Cancer Res..

[61] Pierantoni G.M., Agosti V., Fedele M., Bond H., Caliendo I., Chiappetta G., lo Coco F., Pane F., Turco M.C., Morrone G. (2003). High-mobility group A1 proteins are overexpressed in human leukaemias. Biochem. J..

[62] Takaha N., Sowa Y., Takeuchi I., Hongo F., Kawauchi A., Miki T. (2012). Expression and role of HMGA1 in renal cell carcinoma. J. Urol..

[63] Chuma M., Saeki N., Yamamoto Y., Ohta T., Asaka M., Hirohashi S., Sakamoto M. (2004). Expression profiling in hepatocellular carcinoma with intrahepatic metastasis: Identification of high-mobility group I(Y) protein as a molecular marker of hepatocellular carcinoma metastasis. Keio J. Med..

[64] Sarhadi V.K., Wikman H., Salmenkivi K., Kuosma E., Sioris T., Salo J., Karjalainen A., Knuutila S., Anttila S. (2006). Increased expression of high mobility group A proteins in lung cancer. J. Pathol..

[65] Hillion J., Wood L.J., Mukherjee M., Bhattacharya R., di Cello F., Kowalski J., Elbahloul O., Segal J., Poirier J., Rudin C.M. (2009). Upregulation of MMP-2 by HMGA1 promotes transformation in undifferentiated, large-cell lung cancer. Mol. Cancer Res..

[66] Pomeroy S.L., Tamayo P., Gaasenbeek M., Sturla L.M., Angelo M., McLaughlin M.E., Kim J.Y., Goumnerova L.C., Black P.M., Lau C. (2002). Prediction of central nervous system embryonal tumour outcome based on gene expression. Nature.

[67] Giannini G., Cerignoli F., Mellone M., Massimi I., Ambrosi C., Rinaldi C., Dominici C., Frati L., Screpanti I., Gulino A. (2005). High mobility group A1 is a molecular target for MYCN in human neuroblastoma. Cancer Res..

[68] Giannini G., Cerignoli F., Mellone M., Massimi I., Ambrosi C., Rinaldi C., Gulino A. (2005). Molecular mechanism of HMGA1 deregulation in human neuroblastoma. Cancer Lett..

[69] Cerignoli F., Ambrosi C., Mellone M., Assimi I., di Marcotullio L., Gulino A., Giannini G. (2004). HMGA molecules in neuroblastic tumors. Ann. N Y Acad. Sci..

[70] Liau S.S., Jazag A., Whang E.E. (2006). HMGA1 is a determinant of cellular invasiveness and *in vivo* metastatic potential in pancreatic adenocarcinoma. Cancer Res..

[71] Abe N., Watanabe T., Masaki T., Mori T., Sugiyama M., Uchimura H., Fujioka Y., Chiappetta G., Fusco A., Atomi Y. (2000). Pancreatic duct cell carcinomas express high levels of high mobility group I(Y) proteins. Cancer Res..

[72] Liau S.S., Rocha F., Matros E., Redston M., Whang E. (2008). High mobility group AT-hook 1 (HMGA1) is an independent prognostic factor and novel therapeutic target in pancreatic adenocarcinoma. Cancer.

[73] Takaha N., Hawkins A.L., Griffin C.A., Isaacs W.B., Coffey D.S. (2002). High mobility group protein I(Y): A candidate architectural protein for chromosomal rearrangements in prostate cancer cells. Cancer Res..

[74] Takeuchi I., Takaha N., Nakamura T., Hongo F., Mikami K., Kamoi K., Okihara K., Kawauchi A., Miki T. (2011). High mobility group protein AT-hook 1 (HMGA1) is associated with the development of androgen independence in prostate cancer cells. Prostate.

[75] Rahman M.M., Qian Z.R., Wang E.L., Sultana R., Kudo E., Nakasono M., Hayashi T., Kakiuchi S., Sano T. (2009). Frequent overexpression of HMGA1 and 2 in gastroenteropancreatic neuroendocrine tumours and its relationship to let-7 downregulation. Br. J. Cancer.

[76] Akaboshi S., Watanabe S., Hino Y., Sekita Y., Xi Y., Araki K., Yamamura K., Oshima M., Ito T., Baba H. (2010). HMGA1 is induced by Wnt/β-catenin pathway and maintains cell proliferation in gastric cancer. Am. J. Pathol..

[77] Chiappetta G., Bandiera A., Berlingieri M.T., Visconti R., Manfioletti G., Battista S., Martinez-Tello F.J., Santoro M., Giancotti V., Fusco A. (1995). The expression of the high mobility group HMGI (Y) proteins correlates with the malignant phenotype of human thyroid neoplasias. Oncogene.

[78] Mussnich P., D’Angelo D., Leone V., Croce C.M., Fusco A. (2013). The high mobility group A proteins contribute to thyroid cell transformation by regulating miR-603 and miR-10b expression. Mol. Oncol..

[79] Bandiera A., Bonifacio D., Manfioletti G., Mantovani F., Rustighi A., Zanconati F., Fusco A., di Bonito L., Giancotti V. (1998). Expression of HMGI(Y) proteins in squamous intraepithelial and invasive lesions of the uterine cervix. Cancer Res..

[80] Rajski S.R., Williams R.M. (2000). Observations on the covalent cross-linking of the binding domain (BD) of the high mobility group I/Y (HMG I/Y) proteins to DNA by FR66979. Bioorganice Med. Chem..

[81] Beckerbauer L., Tepe J.J., Cullison J., Reeves R., Williams R.M. (2000). FR900482 class of anti-tumor drugs cross-links oncoprotein HMG I/Y to DNA *in vivo*. Chem. Biol..

[82] Beckerbauer L., Tepe J.J., Eastman R.A., Mixter P.F., Williams R.M., Reeves R. (2002). Differential effects of FR900482 and FK317 on apoptosis, IL-2 gene expression, and induction of vascular leak syndrome. Chem. Biol..

[83] Baron R.M., Lopez-Guzman S., Riascos D.F., Macias A.A., Layne M.D., Cheng G., Harris C., Chung S.W., Reeves R., von Andrian U.H. (2010). Distamycin A inhibits HMGA1-binding to the P-selectin promoter and attenuates lung and liver inflammation during murine endotoxemia. PloS ONE.

[84] Grant M.A., Baron R.M., Macias A.A., Layne M.D., Perrella M.A., Rigby A.C. (2009). Netropsin improves survival from endotoxaemia by disrupting HMGA1 binding to the NOS2 promoter. Biochem. J..

[85] Maasch C., Vater A., Buchner K., Purschke W.G., Eulberg D., Vonhoff S., Klussmann S. (2010). Polyetheylenimine-polyplexes of spiegelmer NOX-A50 directed against intracellular high mobility group protein A1 (HMGA1) reduce tumor growth *in Vivo*. J. Biol. Chem..

[86] Watanabe M., Sheriff S., Lewis K.B., Tinch S.L., Cho J., Balasubramaniam A., Kennedy M.A. (2012). HMGA-targeted phosphorothioate DNA aptamers increase sensitivity to gemcitabine chemotherapy in human pancreatic cancer cell lines. Cancer Lett..

[87] Eilebrecht S., Pellay F., Odenwälder P., Brysbaert G., Benecke B., Benecke A. (2008). EBER2 RNA-induced transcriptome changes identify cellular processes likely targeted during Epstein Barr virus infection. BMC Res. Notes.

[88] Sun J., Lu H., Wang X., Jin H. (2013). MicroRNAs in hepatocellular carcinoma: Regulation, function, and clinical implications. Sci. World J..

[89] Fujita Y., Kuwano K., Ochiya T. (2015). Development of small RNA delivery systems for lung cancer therapy. IJMS.

[90] Fedele M., Fidanza V., Battista S., Pentimalli F., Klein-Szanto A.J., Visone R., de Martino I., Curcio A., Morisco C., del Vecchio L. (2006). Haploinsufficiency of the Hmga1 gene causes cardiac hypertrophy and myelo-lymphoproliferative disorders in mice. Cancer Res..

[91] Carvajal I.M., Baron R.M., Perrella M.A. (2002). High-mobility group-I/Y proteins: Potential role in the pathophysiology of critical illnesses. Crit. Care Med..

[92] Cleynen I., van de Ven W.J. (2008). The HMGA proteins: A myriad of functions (Review). Int. J. Oncol..

[93] Foti D., Chiefari E., Fedele M., Iuliano R., Brunetti L., Paonessa F., Manfioletti G., Barbetti F., Brunetti A., Croce C.M. (2005). Lack of the architectural factor HMGA1 causes insulin resistance and diabetes in humans and mice. Nat. Med..

[94] Bang C., Thum T. (2012). Novel non-coding RNA-based therapeutic approaches to prevent statin-induced liver damage. EMBO Mol. Med..

[95] Archin N.M., Sung J.M., Garrido C., Soriano-Sarabia N., Margolis D.M. (2014). Eradicating HIV-1 infection: Seeking to clear a persistent pathogen. Nat. Publ. Group.

[96] Battistini A., Sgarbanti M. (2014). HIV-1 latency: An update of molecular mechanisms and therapeutic strategies. Viruses.

[97] Chiefari E., Nevolo M.T., Arcidiacono B., Maurizio E., Nocera A., Iiritano S., Sgarra R., Possidente K., Palmieri C., Paonessa F. (2012). HMGA1 is a novel downstream nuclear target of the insulin receptor signaling pathway. Sci. Rep..

[98] Arcidiacono B., Liritano S., Chiefari E., Brunetti S.F., Gu G., Foti D.P., Brunetti A. (2015). Cooperation between HMGA1, PDX-1, and MafA is essential for glucose-induced insulin transcription in pancreatic β cells. Front. Endocrinol. Lausanne.

[99] Garriga J., Graña X. (2014). CDK9 inhibition strategy defines distinct sets of target genes. BMC Res. Notes..

[100] Esposito F., de Martino M., Forzati F., Fusco A. (2014). HMGA1-pseudogene overexpression contributes to cancer progression. Cell Cycle.

